# Effect of Different Mixing Methods on Physicochemical Properties of Mineral Trioxide Aggregate: A Systematic Review

**DOI:** 10.1155/2023/5226095

**Published:** 2023-02-16

**Authors:** Amin Salem Milani, Faraz Radmand, Behrad Rahbani, Mahdi Hadilou, Farnaz Haji Abbas Oghli, Fatemeh Salehnia, Milad Baseri

**Affiliations:** ^1^Endodontic Department, Faculty of Dentistry, Tabriz University of Medical Sciences, Tabriz, Iran; ^2^Dental and Periodontal Research Center, Tabriz University of Medical Sciences, Tabriz, Iran; ^3^School of Dentistry, Shahid Beheshti University of Medical Sciences, Tehran, Iran; ^4^Faculty of Dentistry, Tabriz University of Medical Sciences, Tabriz, Iran; ^5^Student Research Committee, Tabriz University of Medical Sciences, Tabriz, Iran; ^6^Research Center for Evidence-Based Medicine, Tabriz University of Medical Sciences, Tabriz, Iran

## Abstract

**Background:**

Mineral trioxide aggregate (MTA) is a commonly used endodontic biomaterial. The physicochemical properties of MTA have a crucial role in designating clinical outcome, and different factors can affect these properties. Various methods have been used for mixing MTA, including manual, mechanical, and ultrasonic. The aim of this systematic review was to evaluate the effect of different mixing methods on the physicochemical properties of MTA.

**Materials and Methods:**

Electronic databases including PubMed, Embase, Web of Science, and Scopus were searched up to May 2022. In order to cover gray literature, the ProQuest and Google Scholar databases were also searched to detect theses and conference proceedings. For quality assessment of the included studies, we used a modified version of the Cochrane risk of bias tool for randomized controlled trials (RCTs). Experimental studies which had assessed at least one property of MTA and compared at least two different mixing methods of MTA were included in this study. All animal studies, reviews, case reports, and case series were excluded.

**Results:**

Fourteen studies were included. The results showed that the ultrasonic mixing method significantly improved some MTA characteristics, including microhardness, flowability, solubility, setting time, and porosity. However, the mechanical mixing method improved other properties including flowability, solubility, push-out bond strength, and hydration. The manual mixing method showed inferior results compared to other mixing methods in terms of microhardness, flowability, solubility, setting time, push-out bond strength, porosity, and hydration. Different mixing methods had a similar effect on compressive strength, sealing ability, pH and calcium ion release, volume change, film thickness, and flexural strength of MTA.

**Conclusion:**

Mechanical and ultrasonic mixing methods are superior to the manual mixing method in terms of improving physicochemical properties of MTA. No report of selection bias and varieties in methodologies were limitations of evidence.

## 1. Introduction

The role of bioactive materials in dentistry is undeniable. Increasing the usage time of restoration, stimulating the dentin repair process, and favoring adhesive resistance are all positive effects of bioactive materials [1–3]. One of the well-known bioactive materials in the endodontics filed is mineral trioxide aggregate (MTA). MTA is composed of tricalcium silicate, dicalcium silicate, tricalcium aluminate, tetracalcium aluminoferrite, and bismuth oxide [4, 5]. MTA has favorable properties, including bioactivity [6], biocompatibility [7], proper seal in the oral environment [8], excellent marginal adaptation [9, 10], and hard tissue induction capacity [11]. However, it has some disadvantages, including long setting time and difficult handling [12, 13]. Its applications in endodontics are pulp capping, perforation repair, apexification, pulpotomy [14, 15], obturation, and apical plug [16, 17].

Physicochemical properties of an endodontic biomaterial are crucial for their effective clinical use. To attain these ideal characteristics in hydraulic cement, the elements should be completely mixed with water. Three mixing methods commonly used to mix MTA include manual, mechanical, and ultrasonic methods.

Many studies have tried to investigate the effect of different mixing methods on various characteristics of MTA with controversial results. For example, in a survey on flowability, volume change, solubility, and pH of MTA, Duque et al. showed that the mixing methods could not affect the flowability of MTA significantly [18]. However, Shahi et al. [19] showed that the mechanical and ultrasonic mixing methods had higher flowability than the manual technique. The discrepancies in the results of studies may confuse the clinicians for choosing the appropriate way of mixing MTA to achieve optimum physicochemical characteristics. Therefore, this systematic review aimed to compare the effect of different mixing methods on the physicochemical properties of MTA to help clinicians choose the appropriate mixing method.

## 2. Materials and Methods

### 2.1. Study Design

This systematic review was accomplished in agreement with the recommendations of preferred reporting items for systematic reviews and meta-analysis (PRISMA) (Supplementary Material 1) [20, 21].

### 2.2. Search Strategy

As shown in Table 1, the study question was “what are the effects of various MTA mixing methods (*I*) on the physicochemical properties (*O*) of MTA (*P*)?” Electronic databases including PubMed, Embase, Web of Science, and Scopus were searched up to May 2022. In order to cover gray literature, the ProQuest and Google Scholar databases were also searched to detect theses and conference proceedings. Backward and forward reference searching was also performed. The search strategy for all databases is shown in Table 1.

### 2.3. Inclusion Criteria

The inclusion criteria were as follows: in-vitro studies assessing at least one physicochemical property of MTA published in English, and studies comparing at least two different mixing methods with defined sample sizes.

### 2.4. Exclusion Criteria

All animal studies, reviews, case reports, and case series were excluded. Also, studies in which their language was not English were excluded.

### 2.5. Study Selection

After removing duplicates, two authors (A.S.M and F.R) individually screened the titles and abstracts. The full text of the remaining studies was read, and relevant studies according to the eligibility criteria were selected. A third author (M.B) resolved any disagreements between the reviewers.

### 2.6. Data Extraction

Two authors (M.B and F.R) individually extracted the following information from the studies: author(s), year of publication, types of specimens, mixing methods, time of assessment, assessment tools, assessed properties, and outcomes. Any disagreement regarding this process was resolved by a third author (B.R).

### 2.7. Quality Assessment

For quality assessment of the included studies, a modified Cochrane risk of bias tool was used [22, 23]. Two independent reviewers (A.S.M and M.B), which were both dentists and comprehensively informed of the topic and the details of the Cochrane risk of bias tool according to published guidelines [24], checked the following biases: selection bias, detection bias, attrition bias, reporting bias, and other biases. Any disagreements were discussed with a third author (M.H) and resolved.

### 2.8. Assessment of Heterogeneity and Synthesis of Results

The heterogeneity of included studies regarding the mixing method, time of assessment, physicochemical characteristics, type of MTA, and the assessed properties and tests was examined.

## 3. Result

Initially, a total of 1924 papers were identified. After removing duplicates, 1636 papers remained. Next, 1610 articles were excluded after reviewing the abstract and title, and a total of 26 papers remained for full-text assessment. Then, 12 papers were excluded due to the irrelevance of their content. Ten studies focused on placement methods rather than mixing [25–34]. One study investigated other endodontic material [35], and one study investigated packing methods [36].

**Figure 1:**
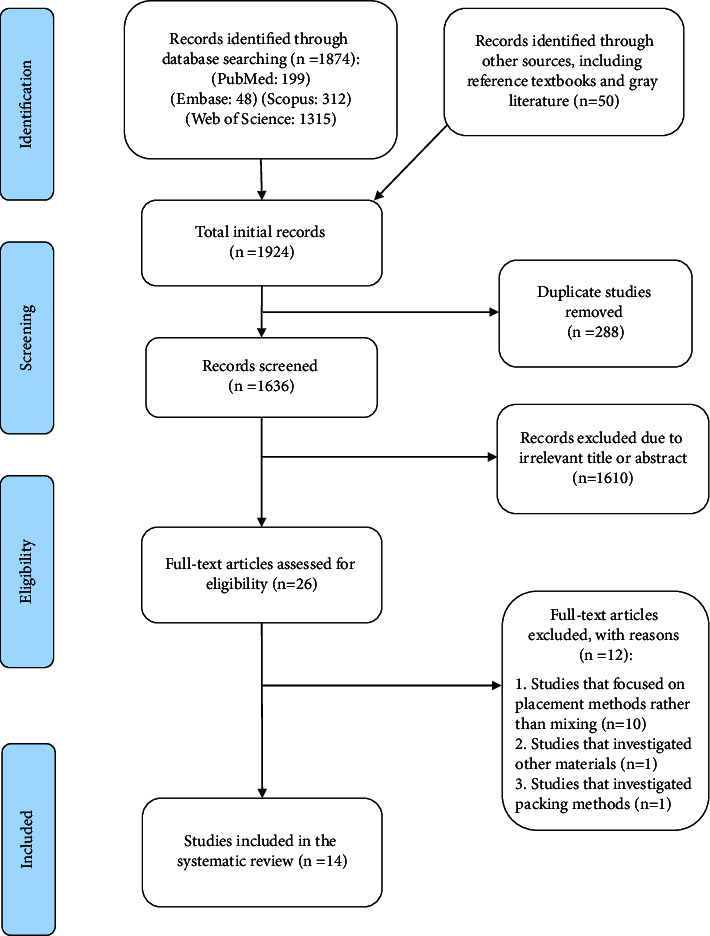
Flow diagram of screening process based on PRISMA protocol.

**Figure 2:**
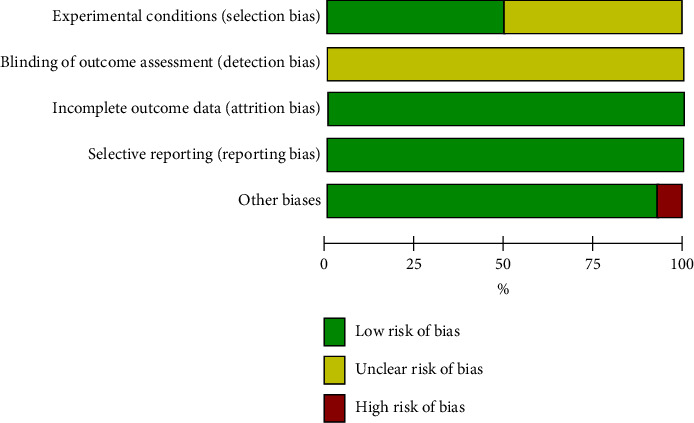
Overall risk of bias.

Finally, 14 papers were included in the review (Figure 1).  Table 2 shows the results of the risk of bias assessment. The risk of bias in the included studies showed low attrition and reporting bias (14/14 studies), followed by other biases (13/14 studies) and selection bias (7/14 studies) (Table 2). The extracted data are summarized in Table 3. All 14 included articles were experimental studies. The summary of the risk of bias evaluation is shown in Figure 2. The main source of bias in the included studies was the detection bias, which was unclear in all articles. Selection bias was also unclear in half of the included studies.

The following characteristics were evaluated in included studies: microhardness (*n* = 2), flowability (*n* = 2), compressive strength (*n* = 2), pH and calcium ion release (*n* = 2), solubility (*n* = 2), initial and final setting time (*n* = 2), film thickness (*n* = 1), volume change (*n* = 2), push-out bond strength (*n* = 2), flexural strength (*n* = 1), porosity (*n* = 3), hydration and phase formation (*n* = 2), and sealing ability (*n* = 2). To unify the terms for mixing methods in this review, the “manual mixing method” was used instead of the hand, conventional, and condensation mixing methods. In addition, the “mechanical mixing method” was used instead of the amalgamator mixing method.

Heterogeneity of the included studies was high regarding the mixing method, time of assessment, physicochemical characteristics, type of MTA, and the assessed properties and tests. The lack of univocal and standard experimental processes made a comparison of the results difficult; therefore, conducting a meta-analysis was not possible.

## 4. Discussion

### 4.1. Microhardness

Microhardness is an indicator of physical characteristics, such as yield strength, modulus of elasticity, tensile strength, and setting [48].

Nekoofar et al. [38] investigated the microhardness of four types of MTA (Angelus white, ProRoot grey, Angelus grey, and ProRoot white) mixed with manual, mechanical, and ultrasonic methods. They showed that irrespective of the type of MTA, the ultrasonic method showed the highest surface microhardness at 4 and 28 days compared with other techniques. Also, no significant difference was found between manual and mechanical methods [38]. The authors attributed better results of the ultrasonic group to the dispersing effect that may provide enough space for water molecules and enhances water diffusion resulting in a better degree of hydration and consequently a greater surface microhardness.

In another study, Saghiri et al. [43] investigated the microhardness of the white MTA mixed with manual, mechanical, and ultrasonic methods. Results showed that the mechanical mixing method had a significantly higher surface hardness compared to the other techniques [43]. They attributed these results to the needle-like crystals in MTA. Interestingly, they attributed the dispersing effect to inferior results obtained in the ultrasonic group. Also, the interaction of needle-like crystals of MTA may reduce the MTA microhardness through interlocking these crystals via ultrasonic energy. However, it should be noted that the growth of crystals takes place gradually after the mixing, and it is unclear how using ultrasonic energy for mixing MTA can affect the interaction of crystals. Therefore, conducting other studies with standard time intervals and various types of MTA cement is needed.

### 4.2. Flowability

Flowability is the ability to penetrate the lateral and accessory canals and irregularities during canal obturation [49]. So, the flowability of the endodontic materials is a critical factor for high-quality obturation.

Shahi et al. [19] investigated the flowability of White ProRoot MTA mixed with manual, mechanical, and ultrasonic methods. They showed that the mechanical and ultrasonic mixing methods had higher flowability than the manual technique [19]. However, mechanical and ultrasonic techniques did not have any significant difference. In the second study, Duque et al. [18] showed that the flowability of MTA was not affected by the mixing technique [18].

The difference between these studies can be attributed to the differences in the details of manual mixing, the type, and the amount of MTA used.

### 4.3. Compressive Strength

The compressive strength is the ability to withstand heavy occlusal and restorative forces [50]. The compressive strength of MTA is affected by factors such as the type of MTA, condensation pressure, mixing method, and the liquid mixed with MTA [8].

Shahi et al. [19] investigated the compressive strength of White ProRoot MTA mixed with manual, mechanical, and ultrasonic methods at two different time intervals (21 hours and 21 days). They showed that the effect of three different mixing methods on compressive strength was not significantly different at any time [19]. In another study, Basturk et al. [39] investigated the compressive strength of ProRoot MTA and MTA Angelus mixed with manual and mechanical mixing methods 4 days after mixing. Irrespective of the MTA type, the mechanical method showed higher compressive strength than the manual method [39]. No significant difference between the two mixing methods in both ProRoot and MTA Angelus was shown. Encapsulation alongside mechanical methods produced more homogeneous MTA slurries [38, 41]. They assumed that better water diffusion might be related to creating a less grainy mixture with fewer unhydrated particles in the mechanical method. Conversely, the manual method was associated with inadequate hydration by restraining the microchannel creation in the material and obstructing the entrance of water molecules to hydrate the material [38]. These conflicting results may be due to difference in the type of MTA used and the time of assessment.

### 4.4. The pH and Calcium Ion Release

As one of the most important features of medical materials is biocompatibility [51], therefore, one of the superiorities of the MTA is its safe use in the dental canal [52]. The biocompatibility of MTA is ascribed to its pH and calcium ion release [53]. Higher pH values are essential for the induction of hard tissue and antimicrobial properties [39, 54–57]. After mixing, the pH of MTA is 10.2 and increases to 12.5 at 3 hours. The authors related the high pH to the continued release of calcium from MTA and the calcium hydroxide formation [58]. Shahi et al. [8] investigated the pH of MTA mixed with manual, mechanical, and ultrasonic methods at the end of the 1^st^ hour. The pH was not significantly affected by different methods. In another study, Duque et al. [18] investigated the pH and the release of calcium ion of MTA Angelus mixed with manual, mechanical, and ultrasonic methods in four different time intervals (3, 24, 72, and 168 hours). The mixing technique did not influence pH values [18]. The calcium ion release was higher with trituration compared to the manual technique at 3 and 168 hours [18].

Collectively, it could be concluded that different mixing methods of MTA did not have a statistically significant effect on pH and the calcium ion release.

### 4.5. Solubility

Solubility is defined as the quantity of a solid material that can be dissolved in a certain amount of solvent. Variations in MTA solubility shown in different studies are due to such factors at the time of immersion, MTA type, and the powder-to-water proportion [13, 59–61]. The low solubility means that the MTA remains where it has been placed, providing satisfactory filling and averting bacterial microleakage [62]. Most studies have suggested low or no solubility for MTA [62–65]. However, a long-term study reported a greater solubility [66]. Shahi et al. [8] investigated the solubility of MTA mixed with manual, mechanical, and ultrasonic methods. The solubility was determined based on the modified ADA guidelines No.30 and ISO 6876 by measuring the weight difference in three different time intervals (1, 7, and 21 days). The mechanical and ultrasonic techniques resulted in higher solubility than the manual technique, though it was not statistically significant [8]. In another study, Duque et al. [18] investigated the solubility of MTA Angelus mixed with manual, mechanical, and ultrasonic methods. The solubility was determined based on the modified ADA specification 57 by measuring the weight difference at the end of day 7. Interestingly, they revealed that the sample's weight was increased over time. The difference in MTA weights in the mechanical and ultrasonic methods was greater compared to the manual method [18].

In conclusion, the results of both studies showed that the weight change in the manual method was smaller than in the ultrasonic and mechanical methods. However, the authors had different interpretations of the weight change of samples, which could be related to the different methodologies used. Duque et al. had not put the samples in the oven prior to weighting to evaporate its water. Therefore, their results showed an increase in weight. The inconsistency in fulfillment of the standard methods of measuring solubility can cause discrepancies in results.

Measuring weight before and after storage in water may not show real solubility since particles of the substance may detach from the cement in the stored environment, or the cement may absorb water. Such interactions seem to mislead investigators in the case of the evaluation of solubility [67, 68]. Further normal saline can be used instead of distilled water to better simulate the physiologic condition of the MTA environment.

### 4.6. Initial and Final Setting Time (ST)

While the initial ST is defined as the time needed by the cement to set and to be rigid enough to bear the lighter Gillmore needle, the final ST is defined as the time necessary for the cement to support the heavier Gilmore needle with no significant indentation [69]. The mixing method, quantity of water used, packing force, and moisture in the environment would affect the ST [70–72]. Although we found three articles conducted on setting and working time, we excluded one of them due to numerous problems in methods and results. In addition, it was not possible to reanalyze the results to find out the exact and correct results [44]. So, two articles about ST were included.

Duque et al. [18] investigated the initial and final ST of MTA Angelus mixed with manual, mechanical, and ultrasonic methods. In 60-second intervals, the mixing methods were not different regarding the initial and final ST of MTA [18].

In the second study, Saghiri et al. [43] investigated the initial ST of white MTA mixed with those mixing methods in 60- or 300-second intervals and concluded that the ultrasonic technique significantly increased the initial ST compared to other techniques [43].

Both studies showed that manual and mechanical methods had not any significant effect on the initial ST of MTA. However, unlike Duque et al. [18], Saghiri et al. [43] exhibited that the ultrasonic technique significantly increased the initial ST. Because their methods were similar, this difference might be attributed to different types of MTA. The only study measuring final ST showed no difference between different methods [18].

### 4.7. Film Thickness

Film thickness (FT) is assessed by placing materials between the two glass slabs for few minutes after mixing based on ISO 6876 : 2001 specifications [44]. Shahi et al. [44] investigated the FT of MTA Angelus mixed with manual, mechanical, and ultrasonic methods 10 minutes after mixing. The mixing method did not influence the FT of MTA [44].

### 4.8. Volume Change

Less volume change during setting would be a favorable characteristic of MTA to assure its adaptation and prevent leakage. Minor expansion might be acceptable by improving the substance's adaptation. However, extreme volume change during the setting process may lead to microleakage, loss of marginal integrity, or fractures and cracks in the dental root [73].

Duque et al. [18] investigated the volume change of MTA Angelus mixed with manual, mechanical, and ultrasonic methods by volumetric micro-CT measurements and reported that at the 7^th^ and 14^th^ days of immersion, there was no significant association between the mixing method and the volume change [18]. In the second study, Shahi et al. [44] investigated the volume change of MTA Angelus mixed with manual, mechanical, and ultrasonic methods by digital Vernier measuring tool at the end of day 30, and they also reported that the volume change of MTA was not affected by the mixing technique [44]. Collectively, both studies confirmed that different methods did not have a significant effect on the volume change of MTA.

### 4.9. Push-Out Bond Strength

One of the superior properties of MTA compared to other materials is the bonding ability to dentin and resistance against displacing forces [74]. Thus, the push-out strength is an important property of MTA as a perforation repair substance and root-end filling material [74–77].

Shahi et al. [41] investigated the push-out bond strength of a 72-hour set MTA Angelus mixed with manual, mechanical, and ultrasonic methods and reported that the mean push-out strength values of MTA by three different methods were similar [41]. In the second study, Uzunoglu et al. [45] investigated the push-out bond strength of ProRoot MTA mixed with manual and mechanical methods. They showed that the mechanical method had significantly higher bond strength in comparison to the manual method. This result was explained by the assumption that the mechanical method creates a less grainy mixture due to better water diffusion [45]. Furthermore, the manual method causes insufficient hydration by restraining microchannel formation inside the MTA [38]. The difference between the results of the above-mentioned studies may be attributed to differences in their methodologies. Uzunoglu et al. [45] did not include the ultrasonic method in the study; they investigated the effect of different moisture conditions on push-out bond strength, which was not investigated in the study by Shahi et al. [41]. Various brands of MTA used in two studies (ProRoot MTA vs. MTA Angelus) might also have an impact on the results.

### 4.10. Flexural Strength

The significance of enhanced flexural strength values in endodontic operations is that it helps the clinicians to use lower amounts of MTA. This feature is important where the space for material placement is limited, and the material should withstand occlusal loading or restorative procedures [78]. The three-point bend test, which is a reliable and valid method, is usually used to evaluate flexural strength [79, 80].

Basturk et al. [40] investigated the flexural strength of white MTA Angelus and white ProRoot MTA mixed with manual and mechanical methods. No significant difference was found between methods [40]. Since there is no data on the effects of the ultrasonic method on the flexural strength of MTA, further studies are needed to draw a definitive conclusion.

### 4.11. Porosity

Porosity is a measure of void spaces within a material. There is a negative correlation between the porosity and flexural strength of MTA [40]. On the other hand, porosity might be beneficial for the MTA hydration process because these pores may provide space for the water to penetrate the material [66].

Basturk et al. [40] investigated the porosity of two types of MTA Angelus and ProRoot MTA mixed with manual and mechanical methods using micro-CT at the end of the 4th day. In the second study, Sisli and Ozbas [47] investigated the porosity of two types of MTA Angelus and ProRoot MTA mixed with manual and mechanical methods using micro-CT at the end of the 7th day. Controversial results were observed concerning mechanical and manual methods, with Sisli and Ozbas [47] reporting higher porosity rates both within the material and at the MTA-dentin interface prepared with the manual method than the mechanical method [47]. Meanwhile, Basturk et al. [40] did not find any significant differences between the same groups [40]. These contrasting results might be explained by different study designs and time of assessments.

In the third study, Ghasemi et al. [42] investigated the porosity of the MTA Angelus mixed with manual and ultrasonic methods using CBCT at the end of the 7^th^ day and reported that ultrasonic mixing results in lower void formation at the MTA-dentin interface than manual method due to the increased flow of the MTA [42]. The increased flow of particles by the ultrasonic method can rearrange particles and displace the voids towards the surface releasing them from the mixture.

In summary, the lack of standard mixing and porosity assessment method makes it difficult to compare the results of different studies to draw a definitive conclusion.

### 4.12. Hydration and Phase Formation

X-ray diffraction analysis is used to assess the hydration and phase formation of MTA. It works by detecting the interferences of monochromatic X-ray beams with the structures present in the material [81] and helps in detecting crystalline particles' formation, their transformations [6], and other various structural parameters [81].

Basturk et al. [46] investigated the hydration and phase formation of tooth-colored ProRoot MTA and White MTA Angelus mixed with manual and mechanical methods at the end of the 4^th^ day and reported that the highest amount of tricalcium silicate, dicalcium silicate, and calcium hydroxide formation in MTA Angelus samples was in those which were mechanically mixed and placed with ultrasonic activation as opposed to manual mixing. These particles are the main crystalline structures associated with MTA hydration [82, 83]. However, they demonstrated no significant differences among ProRoot MTA samples prepared by manual or mechanical methods [46]. The difference between MTA Angelus and ProRoot MTA samples might be attributed to the more homogeneous chemical composition [84, 85] and smaller particle sizes of ProRoot MTA samples resulting in a better wetting of the particles [86], and sample which is less dependent on various mixing methods to ensure hydration. In the second study, Saghiri et al. [43] investigated the hydration and phase formation of the White MTA mixed with manual, mechanical, and ultrasonic methods at three different time intervals (1, 7, and 21 days) and reported that the mechanical method resulted in the highest amount of calcium silicate phases followed by the manual and ultrasonic methods [43].

In summary, it seems that the mechanical method promotes crystallization and phase formation of calcium silicates within MTA by more thorough wetting of particles resulting in a better hydration [43, 46]. Additionally, the mechanical technique prevents the clustering of the powder particles, resulting in more even distribution of particles [43]. Furthermore, direct ultrasonic mixing of the MTA samples can result in higher void formation, which prevents proper crystallization of MTA particles [43].

### 4.13. Sealing Ability

MTA has an excellent sealing ability [9, 10]. Studies have evaluated the effect of different parameters on the sealing ability of MTA [87–89]. One of the parameters which affect sealing ability is the mixing method.

Shahi and Ozbas [37] investigated the bacterial sealing ability of White MTA mixed with manual, mechanical, and ultrasonic methods within 120 days and showed that there was no significant difference in microleakage among the methods [37]. In the second study, Sisli and Ozbas [47] investigated the marginal adaptation of ProRoot MTA and MTA Angelus mixed with manual and mechanical mixing using micro-CT imaging on the 7^th^ day. They considered marginal adaptation as an indicator of sealing ability. They showed that the mechanical method improved the handling characteristics of the MTA, but there was no significant change in marginal adaptation [47]. Collectively, different mixing methods did not have a different effect on the sealing ability of MTA.

### 4.14. Limitations

No report of selection bias and varieties in methodologies were limitations of evidence. Limitations of this review were the lack of clinical trials about the subject which makes it hard to reach a final decision for the clinicians and the lack of studies for each physicochemical characteristic that hardens to definitely interpret the reported results.

## 5. Conclusions

Considering the lack of sufficient studies and heterogeneity of experimental methods, the following conclusions could be made:

1. Ultrasonic mixing has a favorable effect on the MTA characteristics, including microhardness, flowability, solubility, setting time, and porosity. However, this technique might have an unfavorable effect on the hydration phase of MTA.
2. Mechanical mixing method showed favorable effects on some properties of MTA, including flowability, solubility, push-out bond strength, and the hydration. However, setting time might be adversely affected by this method.
3. Manual mixing method showed less favorable effects on microhardness, flowability, solubility, setting time, push-out bond strength, porosity, and hydration compared to mechanical and ultrasonic methods.
4. Finally, regarding the above-mentioned results and noticing that none of the three mixing methods had any superiority on such characteristics as compressive strength, sealing ability, pH and calcium ion release, volume change, film thickness, and flexural strength, it seems that using the manual mixing method is not beneficial for achieving ideal physicochemical properties of MTA. Accordingly, ultrasonic and mechanical mixing methods may help clinicians to achieve satisfactory physicochemical properties. Nonetheless, further investigations are needed to reach more precise and reliable results.

## Figures and Tables

**Table 1:** The study question as PICO and the search strategy.

The parts of PICO	Equals in the study question	The search strategy
P (population)	Mineral trioxide aggregate (MTA)	(((“mineral trioxide aggregate” [Supplementary Concept]) OR ((((((((“MTA cement”[Text Word]) OR “MT aggregate”[Text Word]) OR “MTA-Fillapex”[Text Word]) OR OrthoMTA[Text Word]) OR RetroMTA[Text Word]) OR “aggregate ProRoot”[Text Word]) OR “ProRoot aggregate”[Text Word]) OR “Mineral Trioxide Aggregate”[Text Word])))

I (intervention)	MTA mixing methods	“different mixing method”[Text Word]) OR “different mixing methods”[Text Word]) OR “mixing technique”[Text Word]) OR “mixing techniques”[Text Word]) OR “mixing method”[Text Word]) OR “mixing methods”[Text Word]) OR (“various mixing[Text Word] AND placement technique”[Text Word])) OR (“various mixing[Text Word] AND placement techniques”[Text Word])) OR Trituration[Text Word]) OR condensation[Text Word]) OR ultrasonic[Text Word]) OR “manual mixing”[Text Word]) OR “mechanical mixing”[Text Word]) OR “conventional mixing”[Text Word]) OR “hand mixing”[Text Word]) OR amalgamator[Text Word])

C (comparison)	NA	—

O (outcome)	Physicochemical properties of MTA	Not used in search strategy for not missing articles in order to have access to wide range of papers

NA: Not applicable.

**Table 2:** Assessing the risk of bias in the included studies.

		Experimental conditions (selection bias)	Blinding of outcome assessment (detection bias)	Incomplete outcome data (attrition bias)	Selective reporting (reporting bias)	Other biases
1	Shahi et al. [37]	—	NC	—	—	—
2	Shahi et al. [8]	NC	NC	—	—	—
3	Nekoofar et al. [38]	NC	NC	—	—	—
4	Basturk et al. [39]	—	NC	—	—	—
5	Basturk et al. [40]	NC	NC	—	—	—
6	Shahi et al. [41]	—	NC	—	—	—
7	Shahi et al. [19]	NC	NC	—	—	—
8	Duque et al. [18]	NC	NC	—	—	—
9	Ghasemi et al. [42]	—	NC	—	—	—
10	Saghiri et al. [43]	—	NC	—	—	—
11	Shahi et al. [44]	NC	NC	—	—	+
12	Uzunoglu et al. [45]	—	NC	—	—	—
13	Basturk et al. [46]	NC	NC	—	—	—
14	Sisli and Ozbas [47]	—	NC	—	—	—

NC: Not clear.

**Table 3:** Characteristics of the included studies.

Author & year	Specimen type & (sample size)	Mixing method	Assessment time	Assessed properties & test	Results
Shahi et al. [37]	100 human single-rooted permanent teeth filled with: 1. white MTA (tooth-colored formula, dentsply, Tulsa Dental, Tulsa, OK) (i) ultrasonic (*n* = 15) (ii) conventional (*n* = 15) (iii) amalgamator (*n* = 15) 2. Positive control group (*n* = 5) 3. Negative control group (*n* = 5)	a. Manual b. Mechanical (Amalgamator) c. Ultrasonic	120 days	Bacterial microleakage (Survival time)	1. No significant differences in bacterial microleakage among three mixing methods

Shahi et al. [8]	1. MTA (not mentioned)(*n* = 5)	a. Manual b. Mechanical (Amalgamator) c. Ultrasonic	1 hour 1 day 7 days 21 days	pH (A Metrohm 744 pH meter) Solubility (modified ADA guidelines No. 30)	1. Mixing method had an effect on the solubility of MT with significantly lower solubility with manual mixing method compared to ultrasonic and mechanical mixing methods 2. The pH of MTA was not significantly affected by various mixing methods

Nekoofar et al. [38]	1. ProRoot MTA (white) (dentsply tulsa dental, Johnson City, TN, USA) 2. ProRoot MTA (grey) (dentsply tulsa dental, Johnson City, TN, USA) 3. MTA-Angelus (white) (Angelus Solucoes Odontologicas) 4. MTA-Angelus (grey) (Angelus dental industry products, Londrina, Brazil) (*n* = 35)	a. Mechanical (Amalgamator) b. Manual (condensation) c. Ultrasonic	4 days 28 days	Surface microhardness (Vickers) (European and British Standard (BS EN 843-4:2005))	1. Irrespective of the MTA type, the usage of ultrasonic resulted in the highest surface microhardness at 4 and 28 days, among other mixing methods. Furthermore, no significant difference was found between manual and mechanical methods at both time intervals

Basturk et al. [39]	1. ProRoot MTA (Dentsply Maillefer, Ballaigues, Switzerland) 2. MTA Angelus (Angelus Solucoes Odontologicas, Londrina, Brazil) (*n* = 10)	a. Mechanical (Amalgamator) b. Manual	4 days (37°C, 100% humidity)	Compressive strength (The British Standards Institution)	1. Mechanical mixing technique had higher compressive strength than manual mixing technique

Basturk et al. [40]	1. ProRoot MTA (Dentsply Maillefer, Ballaigues, Switzerland) 2. MTA Angelus (Angelus Solucoes Odontologicas, Londrina, Brazil) (*n* = 10)	a. Mechanical b. Manual	4 days (37 degrees Celsius, 95% humidity)	Flexural strength (3-point bend test) Porosity (micro–computed tomography system SkyScan 1072)	1. No significant differences were found between the mixing methods irrespective of the type of MTA used in flexural strength values 2. No significant porosity differences were identified between different mixing groups

Shahi et al. [41]	Single-rooted human teeth filled with: MTA Angelus (Angelus Dental Industry Products, Londrina, Brazil) (*n* = 20)	a. Mechanical (Amalgamator) b. Manual c. Ultrasonic	3 days	Push-out bond strength	1. The results showed that there was not a significant difference between the three groups regarding the mean push-out strength values

Shahi et al. [19]	White ProRoot MTA (dentsply, tulsa dental, Tulsa, OK, USA) (*n* = 6)	a. Manual b. Mechanical (Amalgamator) c. Ultrasonic	10 minutes 21 hours 21 days	Flowability (The flowability of the materials was tested according to the ISO 6876 criteria) Compressive strength (Compressive strength of the samples was measured using ISO 6876 guidelines)	1. The mechanical and manual techniques exhibited the highest and lowest flowability, respectively 2. The effect of different mixing techniques on compressive strength was not significant

Duque et al. [18]	MTA (Angelus, Londrina, Parana, Brazil) (*n* = 10)	a. Manual b. Mechanical (Amalgamator) c. Ultrasonic	10 minutes — 7 days 7 days 14 days 3 hours 24 hours 72 hours 168 hours 3 hours 24 hours 72 hours 168 hours	Flowability (ANSI/ADA specification No. 57/2012) Setting time (ASTM C266-08) Solubility (ADA Specification 57) Volume change (Volumetric micro-CT measurements) pH pH meter with a precision of 0.01 Calcium release atomic absorption spectrophotometer	1. The flowability of MTA was not affected by the mixing technique 2. The effect of different mixing techniques on initial and final setting times was not significant 3. No significant differences regarding volume change were found between the groups 4. No significant differences regarding solubility were found between the groups 5. No significant differences regarding pH and calcium ion release were found between the groups

Ghasemi et al. [42]	Human maxillary central incisors filled with: 1. MTA (Angelus, Londrina, Paraná, Brazil) MTA was mixed with a 3 : 1 powder to water ratio in all the studied groups. (*n* = 20)	a. Manual b. Ultrasonic	7 days (37°C, 100% humidity)	Porosity (Void counts and Void dimensions)	1. Ultrasonic mixing resulted in less void formation at the MTA-dentin interface than manual mixing

Saghiri et al. [43]	1. WMTA (Dentsply;Tulsa Dental, Tulsa, OK, USA) (*n* = 7)	a. Manual b. Mechanical (Amalgamator) c. Ultrasonic	1 day 7 days 21 days	Microhardness (Vickers) Setting time (ISO 6876 : 2001) Hydration and phase formation (X-ray diffraction (XRD))	1. Mechanical mixing technique had a significantly higher microhardness compared to the other two mixing techniques 2. Ultrasonic mixing technique significantly increased the initial setting time in comparison with other mixing methods 3. Mechanical mixing method showed the highest amount of calcium silicate phases compared to other mixing methods

Shahi et al. [44]	MTA (Angelus, Londrina, Paraná, Brazil) (*n* = 6)	a. Manual b. Mechanical (Amalgamator) c. Ultrasonic	—	Setting time (ISO 6786 : 2001 specification) Working time (ISO 6876 : 2001) Dimensional changes (ISO 6876 : 2001) Film thickness (ISO 6876 : 2001)	1. The different mixing methods had no significant effect on the dimensional change and film thickness of MTA

Uzunoglu et al. [45]	1. ProRoot MTA (Dentsply Tulsa Dental, Tulsa, OK, USA) (*n* = 20)	a. Mechanical (Amalgamator) b. Manual	—	Push-out test	1. Mechanical mixing method showed significantly better bond strength values compared to the manual group

Basturk et al. [46]	1. Tooth-colored ProRoot MTA (Dentsply Maillefer, Ballaigues, Switzerland) 2. White MTA Angelus (Angelus Soluçoes Odontologicas, Londrina, Brazil) (*n* = 4)	a. Manual b. Mechanical (Amalgamator)	4 days (37°C, 100% humidity)	Hydration and Phase formation (X-ray diffraction (XRD))	1. For ProRoot MTA, the mixing and placement techniques did not cause any significant difference 2. For MTA Angelus, the mechanical mixing method combined with the ultrasonic placement method improved the calcium hydroxide phase formation

Nihal Sisli et al. [47]	1. Pro Root MTA (Dentsply Maillefer, Ballaigues, Switzerland) 2. MTA Angelus (Solucoes Odontologicas, Londrina, Brazil) (*n* = 15)	a. Manual b. Mechanical (Amalgamator)	7 days	Sealing ability Porosity (micro-CT imaging)	1. The mechanically mixed MTA showed significantly lower porosity than the manually mixed groups 2. There was no significant difference in the sealing ability among the groups

## Data Availability

The data used to support the findings of this study are available from the corresponding author upon request.

## Supplementary Materials

Supplementary Materials Supplementary Materials 1. PRISMA checklist.

## References

[1] Lardani L., Derchi G., Marchio V., Carli E. (2022). One-year clinical performance of Activa™ bioactive-restorative composite in primary molars. *Children*.

[2] Ladino L. G., Bernal A., Calderón D., Cortés D. (2021). Bioactive materials in restorative dentistry: a literature review. *Science*.

[3] Cianetti S., Abraha I., Pagano S., Lupatelli E., Lombardo G. (2018). Sonic and ultrasonic oscillating devices for the management of pain and dental fear in children or adolescents that require caries removal: a systematic review. *BMJ Open*.

[4] Rahimi S., Shahi S., Lotfi M., Yavari H. R., Charehjoo M. E. (2008). Comparison of microleakage with three different thicknesses of mineral trioxide aggregate as root-end filling material. *Journal of Oral Science*.

[5] Camilleri J. (2009). Evaluation of selected properties of mineral trioxide aggregate sealer cement. *Journal of Endodontics*.

[6] Akhavan H., Mohebbi P., Firouzi A., Noroozi M. (2016). X-Ray diffraction analysis of ProRoot mineral trioxide aggregate hydrated at different pH values. *Iranian Endodontic Journal*.

[7] Koh E. T., McDonald F., Pitt Ford T. R., Torabinejad M. (1998). Cellular response to mineral trioxide aggregate. *Journal of Endodontics*.

[8] Shahi S., Ghasemi N., Rahimi S. (2015). The effect of different mixing methods on the pH and solubility of mineral trioxide aggregate and calcium-enriched mixture. *Iranian Endodontic Journal*.

[9] Torabinejad M., Watson T., Pitt Ford T. (1993). Sealing ability of a mineral trioxide aggregate when used as a root end filling material. *Journal of Endodontics*.

[10] Torabinejad M., Higa R. K., McKendry D. J., Pitt Ford T. R. (1994). Dye leakage of four root end filling materials: effects of blood contamination. *Journal of Endodontics*.

[11] Apaydin E. S., Shabahang S., Torabinejad M. (2004). Hard-tissue healing after application of fresh or set MTA as root-end-filling material. *Journal of Endodontics*.

[12] Asgary S., Shahabi S., Jafarzadeh T., Amini S., Kheirieh S. (2008). The properties of a new endodontic material. *Journal of Endodontics*.

[13] Islam I., Kheng Chng H., Jin Yap A. U. (2006). Comparison of the physical and mechanical properties of MTA and Portland cement. *Journal of Endodontics*.

[14] Maroto M., Barberia E., Vera V., Garcia-Godoy F. (2007). Mineral trioxide aggregate as pulp dressing agent in pulpotomy treatment of primary molars: 42-month clinical study. *American Journal of Dentistry*.

[15] Cardoso-Silva C., Barbería E., Maroto M., García-Godoy F. (2011). Clinical study of Mineral Trioxide Aggregate in primary molars. Comparison between Grey and White MTA—a long term follow-up (84 months). *Journal of Dentistry*.

[16] Prati C., Gandolfi M. G. (2015). Calcium silicate bioactive cements: biological perspectives and clinical applications. *Dental Materials*.

[17] Pace R., Giuliani V., Nieri M., Di Nasso L., Pagavino G. (2014). Mineral trioxide aggregate as apical plug in teeth with necrotic pulp and immature apices: a 10-year case series. *Journal of Endodontics*.

[18] Duque J. A., Fernandes S. L., Bubola J. P., Duarte M. A. H., Camilleri J., Marciano M. A. (2018). The effect of mixing method on tricalcium silicate‐based cement. *International Endodontic Journal*.

[19] Shahi S., Ghasemi N., Rahimi S. (2015). The effect of different mixing methods on the flow rate and compressive strength of mineral trioxide aggregate and calcium-enriched mixture. *Iranian Endodontic Journal*.

[20] Moher D., Liberati A., Tetzlaff J., Altman D. G. (2009). preferred reporting items for systematic reviews and meta-analyses: the PRISMA statement. *Journal of Clinical Epidemiology*.

[21] Shamseer L., Moher D., Clarke M. (2015). Preferred reporting items for systematic review and meta-analysis protocols (PRISMA-P) 2015: elaboration and explanation. *BMJ*.

[22] Higgins J. P. T., Altman D. G., Gøtzsche P. C. (2011). The Cochrane Collaboration’s tool for assessing risk of bias in randomised trials. *BMJ*.

[23] Koletsi D., Iliadi A., Eliades T., Eliades G. (2019). In vitro simulation and in vivo assessment of tooth wear: a meta-analysis of in vitro and clinical research. *Materials*.

[24] Lundh A., Gøtzsche P. C. (2008). Recommendations by Cochrane Review Groups for assessment of the risk of bias in studies. *BMC Medical Research Methodology*.

[25] Keleş A., Torabinejad M., Keskin C., Sah D., Uzun İ, Alçin H. (2018). Micro-CT evaluation of voids using two root filling techniques in the placement of MTA in mesial root canals of Vertucci type II configuration. *Clinical Oral Investigations*.

[26] Küçükkaya Eren S., Aksel H., Askerbeyli Örs S. (2019). Obturation quality of calcium silicate-based cements placed with different techniques in teeth with perforating internal root resorption: a micro-computed tomographic study. *Clinical Oral Investigations*.

[27] Küçükkaya Eren S., Aksel H., Serper A. (2016). Effect of placement technique on the push-out bond strength of calcium-silicate based cements. *Dental Materials Journal*.

[28] Alcalde M. P., Vivan R. R., Marciano M. A. (2018). Effect of ultrasonic agitation on push-out bond strength and adaptation of root-end filling materials. *Restorative dentistry & endodontics*.

[29] Aminoshariae A., Hartwell G. R., Moon P. C. (2003). Placement of mineral trioxide aggregate using two different techniques. *Journal of Endodontics*.

[30] Donyavi Z., Khoshbin E., Esmaeilzadeh M., Rezaei-Soufi L., Kermani N. (2017). Microleakage of two root-end filling materials in the cavities prepared by laser and ultrasonic technique: an in-vitro study. *Italian Journal of Vascular and Endovascular Surgery*.

[31] Friedl C. C., Williamson A. E., Dawson D. V., Gomez M. R., Liu W. (2016). Comparison of mechanical and indirect ultrasonic placement technique on mineral trioxide aggregate retrofill density in simulated root-end surgery. *Journal of Endodontics*.

[32] Mandava P., Bolla N., Thumu J., Vemuri S., Chukka S. (2015). Microleakage evaluation around retrograde filling materials prepared using conventional and ultrasonic techniques. *Journal of Clinical and Diagnostic Research: Journal of Clinical and Diagnostic Research*.

[33] Aksel H., Arslan E., Puralı N., Uyanık Ö, Nagaş E. (2019). Effect of ultrasonic activation on dentinal tubule penetration of calcium silicate‐based cements. *Microscopy Research and Technique*.

[34] Basturk F. B., Nekoofar M. H., Gunday M., Dummer P. M. (2015). Effect of varying water-to-powder ratios and ultrasonic placement on the compressive strength of mineral trioxide aggregate. *Journal of Endodontics*.

[35] Lopes F. C., Zangirolami C., Mazzi-Chaves J. F. (2019). Effect of sonic and ultrasonic activation on physicochemical properties of root canal sealers. *Journal of Applied Oral Science: Revista FOB*.

[36] Oraie E., Ghassemi A. R., Eliasifar G., Sadeghi M., Shahravan A. (2012). Apical sealing ability of MTA in different liquid to powder ratios and packing methods. *Iranian Endodontic Journal*.

[37] Shahi S., Bashirzadeh A., Yavari H. R. (2017). Effect of different mixing methods on the bacterial microleakage of white Portland cement and white Mineral Trioxide Aggregate. *Journal of Dental Research, Dental Clinics, Dental Prospects*.

[38] Nekoofar M. H., Aseeley Z., Dummer P. M. H. (2010). The effect of various mixing techniques on the surface microhardness of mineral trioxide aggregate. *International Endodontic Journal*.

[39] Basturk F. B., Nekoofar M. H., Günday M., Dummer P. M. (2013). The effect of various mixing and placement techniques on the compressive strength of mineral trioxide aggregate. *Journal of Endodontics*.

[40] Basturk F. B., Nekoofar M. H., Gunday M., Dummer P. M. (2014). Effect of various mixing and placement techniques on the flexural strength and porosity of mineral trioxide aggregate. *Journal of Endodontics*.

[41] Shahi S., Rahimi S., Yavari H. R. (2012). Effects of various mixing techniques on push-out bond strengths of white mineral trioxide aggregate. *Journal of Endodontics*.

[42] Ghasemi N., Janani M., Razi T., Atharmoghaddam F. (2017). Effect of different mixing and placement methods on the quality of MTA apical plug in simulated apexification model. *Journal of clinical and experimental dentistry*.

[43] Saghiri M. A., Garcia‐Godoy F., Gutmann J. L., Lotfi M., Asatourian A. (2014). Effects of various mixing techniques on physical properties of W hite M ineral T rioxide A ggregate. *Dental Traumatology*.

[44] Shahi S., Ghasemi N., Rahimi S. (2015). The effect of different mixing methods on working time, setting time, dimensional changes and film thickness of mineral trioxide aggregate and calcium-enriched mixture. *Iranian Endodontic Journal*.

[45] Uzunoglu E., Aktemur Turker S., Uyanik M. O., Nagas E. (2016). Effects of mixing techniques and dentin moisture conditions on push-out bond strength of ProRoot MTA and Biodentine. *Journal of Adhesion Science and Technology*.

[46] Basturk F. B., Nekoofar M. H., Gunday M., Dummer P. M. H. (2018). X-ray diffraction analysis of MTA mixed and placed with various techniques. *Clinical Oral Investigations*.

[47] Sisli S. N., Ozbas H. (2017). Comparative micro–computed tomographic evaluation of the sealing quality of ProRoot MTA and MTA Angelus apical plugs placed with various techniques. *Journal of Endodontics*.

[48] Salem Milani A., Banifatemeh A., Rahimi S., Jafarabadi M. A. (2015). The effect of using propylene glycol as a vehicle on the microhardness of mineral trioxide aggregate. *General Dentistry*.

[49] Rocha BdC. S., Limoeiro A. G. d S., Bueno C. E. d S., Souza F. S. d, Braitt A. H. (2017). Estudo in vitro do nível de escoamento de cinco cimentos endodônticos: e. *Dental Press Endod*.

[50] Ghasemi N., Rahimi S., Shahi S., Salem Milani A., Rezaei Y., Nobakht M. (2016). Compressive strength of mineral trioxide aggregate with propylene glycol. *Iranian Endodontic Journal*.

[51] Pagano S., Coniglio M., Valenti C. (2020). Biological effects of Cannabidiol on normal human healthy cell populations: systematic review of the literature. *Biomedicine & Pharmacotherapy*.

[52] Torabinejad M., Parirokh M. (2010). Mineral trioxide aggregate: a comprehensive literature review—part II: leakage and biocompatibility investigations. *Journal of Endodontics*.

[53] Sarkar N., Caicedo R., Ritwik P., Moiseyeva R., Kawashima I. (2005). Physicochemical basis of the biologic properties of mineral trioxide aggregate. *Journal of Endodontics*.

[54] Massi S., Tanomaru-Filho M., Silva G. F. (2011). pH, calcium ion release, and setting time of an experimental mineral trioxide aggregate–based root canal sealer. *Journal of Endodontics*.

[55] Estrela C., Bammann L. L., Estrela C. RdA., Silva R. Sd, Pecora J. D. (2000). Antimicrobial and Chemical Study of MTA, Portland Cement, Calcium Hydroxide Paste Sealapex and Dycal. *Braz Dent J*.

[56] Duarte M. A. H., Demarchi A. C. C. d O., Yamashita J. C., Kuga M. C., Fraga S. C. (2003). pH and calcium ion release of 2 root-end filling materials. *Oral Surgery, Oral Medicine, Oral Pathology, Oral Radiology & Endodontics*.

[57] Vivan R. R., Zapata R. O., Zeferino M. A. (2010). Evaluation of the physical and chemical properties of two commercial and three experimental root-end filling materials. *Oral Surgery, Oral Medicine, Oral Pathology, Oral Radiology & Endodontics*.

[58] Parirokh M., Torabinejad M. (2010). Mineral trioxide aggregate: a comprehensive literature review—part I: chemical, physical, and antibacterial properties. *Journal of Endodontics*.

[59] Fridland M., Rosado R. (2003). Mineral trioxide aggregate (MTA) solubility and porosity with different water-to-powder ratios. *Journal of Endodontics*.

[60] Reyes-Carmona J. F., Felippe M. S., Felippe W. T. (2009). Biomineralization ability and interaction of mineral trioxide aggregate and white portland cement with dentin in a phosphate-containing fluid. *Journal of Endodontics*.

[61] Bodanezi A., Carvalho N., Silva D. (2008). Immediate and delayed solubility of mineral trioxide aggregate and Portland cement. *Journal of Applied Oral Science*.

[62] Danesh G., Dammaschke T., Gerth H. U. V., Zandbiglari T., Schäfer E. (2006). A comparative study of selected properties of ProRoot mineral trioxide aggregate and two Portland cements. *International Endodontic Journal*.

[63] Torabinejad M., Hong C., McDonald F., Pittford T. (1995). Physical and chemical properties of a new root-end filling material. *Journal of Endodontics*.

[64] Poggio C., Lombardini M., Alessandro C., Simonetta R. (2007). Solubility of root-end–filling materials: a comparative study. *Journal of Endodontics*.

[65] Shie M.-Y., Huang T.-H., Kao C.-T., Huang C.-H., Ding S.-J. (2009). The effect of a physiologic solution pH on properties of white mineral trioxide aggregate. *Journal of Endodontics*.

[66] Fridland M., Rosado R. (2005). MTA solubility: a long term study. *Journal of Endodontics*.

[67] Örstavik D. (1983). Weight loss of endodontic sealers, cements and pastes in water. *European Journal of Oral Sciences*.

[68] Kazemi R. B., Safavi K. E., Spångberg L. S. (1993). Dimensional changes of endodontic sealers. *Oral Surgery, Oral Medicine, Oral Pathology*.

[69] Gandolfi M. G., Iacono F., Agee K. (2009). Setting time and expansion in different soaking media of experimental accelerated calcium-silicate cements and ProRoot MTA. *Oral Surgery, Oral Medicine, Oral Pathology, Oral Radiology & Endodontics*.

[70] Ber B. S., Hatton J. F., Stewart G. P. (2007). Chemical modification of ProRoot MTA to improve handling characteristics and decrease setting time. *Journal of Endodontics*.

[71] Cavenago B. C., Pereira T. C., Duarte M. A. H. (2014). Influence of powder‐to‐water ratio on radiopacity, setting time, pH, calcium ion release and a micro‐CT volumetric solubility of white mineral trioxide aggregate. *International Endodontic Journal*.

[72] Charland T., Hartwell G. R., Hirschberg C., Patel R. (2013). An evaluation of setting time of mineral trioxide aggregate and EndoSequence root repair material in the presence of human blood and minimal essential media. *Journal of Endodontics*.

[73] Camilleri J., Grech L., Galea K. (2014). Porosity and root dentine to material interface assessment of calcium silicate-basedroot-end filling materials. *Clinical Oral Investigations*.

[74] Salem Milani A., Froughreyhani M., Charchi Aghdam S., Pournaghiazar F., Asghari Jafarabadi M. (2013). Mixing with propylene glycol enhances the bond strength of mineral trioxide aggregate to dentin. *Journal of Endodontics*.

[75] Goracci C., Tavares A. U., Fabianelli A. (2004). The adhesion between fiber posts and root canal walls: comparison between microtensile and push‐out bond strength measurements. *European Journal of Oral Sciences*.

[76] Ghasemi N., Reyhani M. F., Salem Milani A., Mokhtari H., Khoshmanzar F. (2016). Effect of calcium hydroxide on the push-out bond strength of endodontic biomaterials in simulated furcation perforations. *Iranian Endodontic Journal*.

[77] Rahimi S., Ghasemi N., Shahi S. (2013). Effect of blood contamination on the retention characteristics of two endodontic biomaterials in simulated furcation perforations. *Journal of Endodontics*.

[78] Walker M. P., Diliberto A., Lee C. (2006). Effect of setting conditions on mineral trioxide aggregate flexural strength. *Journal of Endodontics*.

[79] Milani A. S., Banifatemeh A., Reyhani M. F., Rahimi S., Zand V. (2017). The effect of using propylene glycol as a vehicle on the flexural strength of mineral trioxide aggregate. *International Journal of Clinical Dentistry*.

[80] Moghaddam N., Jokandan M. E., Nouri-Vaskeh M., Milani A. S. (2018). Comparison of flexural strength of mineral trioxide aggregate, calcium-enriched mixture and BioAggregate. *Iranian Endodontic Journal*.

[81] Bunaciu A. A., UdriŞTioiu E. G., Aboul-Enein H. Y. (2015). X-ray diffraction: instrumentation and applications. *Critical Reviews in Analytical Chemistry*.

[82] Belío-Reyes I. A., Bucio L., Cruz-Chavez E. (2009). Phase composition of ProRoot mineral trioxide aggregate by X-ray powder diffraction. *Journal of Endodontics*.

[83] Islam I., Chng H. K., Yap A. U. J. (2006). X‐ray diffraction analysis of mineral trioxide aggregate and Portland cement. *International Endodontic Journal*.

[84] Song J.-S., Mante F. K., Romanow W. J., Kim S. (2006). Chemical analysis of powder and set forms of Portland cement, gray ProRoot MTA, white ProRoot MTA, and gray MTA-Angelus. *Oral Surgery, Oral Medicine, Oral Pathology, Oral Radiology & Endodontics*.

[85] Dammaschke T., Gerth H. U., Züchner H., Schäfer E. (2005). Chemical and physical surface and bulk material characterization of white ProRoot MTA and two Portland cements. *Dental Materials*.

[86] Komabayashi T., Spångberg L. S. (2008). Comparative analysis of the particle size and shape of commercially available mineral trioxide aggregates and Portland cement: a study with a flow particle image analyzer. *Journal of Endodontics*.

[87] Vosoughhosseini S., Lotfi M., Shahmoradi K. (2011). Microleakage comparison of glass-ionomer and white mineral trioxide aggregate used as a coronal barrier in nonvital bleaching. *Medicina Oral, Patología Oral y Cirugía Bucal*.

[88] Mohammad F., Amin S. M., Saeed R., Sahar S., Somaieh F. (2011). Comparison of apical sealing ability of resected mineral trioxide aggregate, gutta-percha and a resin-based root canal filling material (resilon). *African Journal of Biotechnology*.

[89] Lotfi M., Vosoughhosseini S., Saghiri M. (2011). *Medicina Oral, Patología Oral y Cirugía Bucal*.

